# Nanoetched Stainless Steel Architecture Enhances Cell Uptake of Biomacromolecules and Alters Protein Corona Abundancy

**DOI:** 10.1021/acsami.4c14492

**Published:** 2024-10-17

**Authors:** Thomas Pho, Maeve A. Janecka, Samantha M. Pustulka, Julie A. Champion

**Affiliations:** † School of Chemical and Biomolecular Engineering, Georgia Institute of Technology, 950 Atlantic Dr. NW, Atlanta, Georgia 30332-2000, United States; ‡ BioEngineering Program, Georgia Institute of Technology, Atlanta, Georgia 30332, United States

**Keywords:** Nanostructure, Cell adhesion, Intracellular delivery, Stainless steel, Protein corona

## Abstract

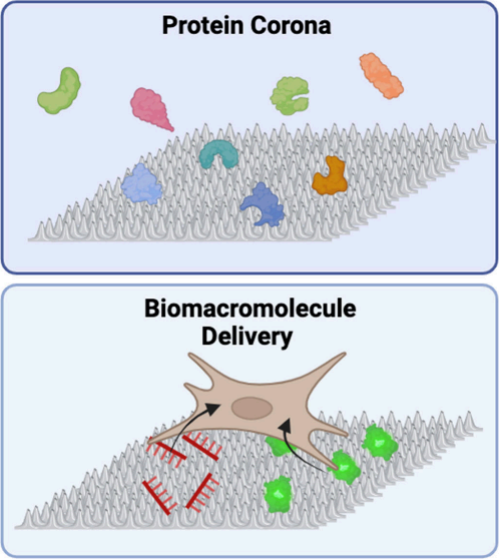

Nanotexture on biocompatible surfaces promotes cell adhesion and proliferation. High aspect ratio nanoachitecture serves as an ideal interface between implant materials and host cells that is well-suited for localized therapeutic delivery. Despite this potential, nanotextured surfaces have not been widely applied for biomacromolecule delivery. Here, we employed a low-cost, industrially relevant nanoetching process to modify the surface of biocompatible stainless steel 316 (SS316L), creating nanotextured SS316L (NT-SS316L) as a material for intracellular biomacromolecule delivery. As biomacromolecule cargoes are adsorbed to the steel and ultimately would be used in protein-rich environments, we performed serum protein corona analysis on unmodified SS316L and NT-SS316L using tandem mass spectrometry. We observed an increase in proteins associated with cell adhesion on the surface of NT-SS316L compared to that of SS316L, supporting literature reports of enhanced adhesion on nanotextured materials. For delivery to adherent cells, a “hard corona” of model biomacromolecule cargoes including superfolder green fluorescent protein (sfGFP) charge variants, cytochrome c, and siRNA was adsorbed on NT-SS316L to assess delivery. Nanotextured surfaces enhanced cellular biomacromolecule uptake and delivered cytosolic-functional proteins and nucleic acids through energy-dependent endocytosis. Collectively, these findings indicate that NT-SS316L holds potential as a surface modification for implants to achieve localized drug delivery for a variety of biomedical applications.

## 1 Introduction

Clinical need for dental implants, orthopedic implants, and stents is increasing rapidly.^1,2^ For example, prosthetic joint arthroplasties are projected to increase to 3.8 million procedures per year by 2030.^3^ Medical implants are often made from metallic materials due to their desirable physical properties, such as mechanical strength and durability.^4^ Electropolished stainless steel 316L (SS316L) is a commonly used implant material due to its high corrosion resistance toward biological fluids, biocompatibility, and low-cost fabrication.^5^ SS316L is also noted to have equal or superior biomechanical properties compared to titanium for medical implants, although titanium plates have a lower rate of surgical failure.^6^ One of the current limitations of metallic implants is the risk of serious infection, morbidity, and mortality when delayed healing occurs postsurgery.^7^ To improve wound healing, injectable growth factors have been used to promote revascularization and new bone growth at surgical implant sites. However, these strategies typically require biomaterials, such as hydrogels, for sufficient retention of bioactive protein at the healing site.^8^ Medical grade SS316L implant surfaces have been previously employed as drug delivery platforms, as the enrichment of drugs onto implant materials offers localized delivery in sites of interest, reduces inflammatory responses, and promotes regeneration.^9−13^ Several studies have used stainless steel implants coated with small, anti-inflammatory molecules such as estradiol, everolimus, and ibuprofen to promote cellular proliferation and reduce inflammation *in vitro* and *in vivo*.^11,12,14^ However, larger biomacromolecules with intracellular targets have been more difficult to integrate for delivery due to challenges with low cell membrane permeability.^15^ Currently, there is an unmet need for low-cost localized delivery of exogenous biomolecules to implant sites for improved healing, integration, and regeneration.

Nanotextured materials are of particular interest due to their direct interfacial interaction with cell membranes. Spike-like architectures of 10 to 1000 nm have been shown to modify cellular behaviors, such as adhesion and protein absorption, in ways that are favorable for wound healing.^16−19^ Several studies that used nanostructured hybrid titanium or SS316L surfaces demonstrated enhanced osteoblast attachment, adhesion, well-spread cellular morphology and organized action fibers on nanotextured surfaces compared to smooth surfaces, making nanotextured architecture a promising topographic modification for implants.^19,20^ However, scalable manufacturing of nanotexture architecture has been limited as often the fabrication methods are expensive and labor-intensive, such as reactive ion etching and silicon lithography.^21−23^ Additionally, while many studies have generated nanostructures on metallic materials to improve cell proliferation and adhesion, the use of nanostructures as drug delivery platforms for exogenous biomacromolecules has few examples.^20,23^ Arrays of porous silicon nanoneedles with micron lengths produced by lithography demonstrated intracellular delivery of proteins, nucleic acids and quantum dots entrapped in the pores *in vitro* and intradermally *in vivo* due to direct penetration into the cytosol.^21,23^ Recently, electrodeposited gold nanoneedles on glass were demonstrated to increase delivery of soluble proteins, siRNA and quantum dots to adherent cells *in vitro*.^20^ Proteomic analysis of cells indicated the delivery mechanism to be cell cycle arrest in the G2 phase, when endocytic processes are most frequent. Altogether, the nanotexture literature supports the potential of biomacromolecule delivery using nanotextured stainless steel, which could be created directly on implant surfaces to promote healing and regeneration.

Several techniques have been used to immobilize functional biomacromolecules onto steel surfaces. For example, the covalent immobilization of enzymes has been achieved on stainless steel surfaces using branched poly(ethylene imine) or glutaraldehyde for binding and cross-linking.^10^ The steel-protein interface can host proteins such as lysozyme while enabling their antibacterial enzymatic activity.^10^ Noncovalent formation of a stable primary adsorbed layer was achieved by flowing monoclonal antibodies over steel in a flow reactor.^24^ Protein adsorption phenomena on nanotextured biomaterials are critical to understand, both for their potential to affect downstream biological functions and immune interactions due to the biomaterial’s “protein fingerprint”, or protein corona, and for therapeutic biomacromolecule loading of nanotextured biomaterials.^25^ Several studies have examined protein adsorption behavior on stainless steel surfaces at different temperatures and flow regimes.^24,26^ However, a comprehensive analysis of protein adsorption on nanotextured stainless steel has not been performed. Surface protein coronas consist of two distinctive phases: soft and hard. While the soft corona has a weak association to the surface and can be easily washed away, the hard corona is strongly adsorbed and is a stable coating. Manipulating the hard corona is a promising strategy for interfacial drug delivery, as it enables cargo to have direct contact with biological interfaces. For example, an apolipoprotein E corona applied to nanoparticles has been used for drug delivery to the brain.^27^ Using the “hard corona” strategy on inorganic nanotextured surfaces is a promising technique for intracellular drug delivery.

In this study, nanotextured SS316L (NT-SS316L) was used as a metallic scaffold for the delivery of various biomacromolecules loaded onto a hard corona. We previously developed a low-cost, scalable electrochemical method of fabricating NT-SS316L.^28^ NT-SS316L was determined to be antibacterial to both Gram-positive and Gram-negative bacteria while noncytotoxic to fibroblasts and maintains the corrosion resistance of unmodified SS316L. These properties make nanotextured stainless steel a promising candidate for implants, with or without localized delivery of biotherapeutics. In the body, stainless steel implants experience protein fouling analogous to the protein corona that forms on nanoparticles.^29^ The adsorption of biomolecules can initiate thrombosis, facilitate recognition by the mononuclear phagocytic system, and increase undesired cell attachment, causing detrimental effects or implant failure.^30^ We evaluated the NT-SS316L protein corona from serum proteins using liquid chromatography–tandem mass spectrometry (LC–MS/MS) and bioinformatics.^31^ Then, we created a hard protein corona coating of selected biomacromolecules to assess the ability of NT-SS316L to cytosolically deliver functional protein and siRNA. We demonstrated that NT-SS316L increased intracellular delivery of cargoes compared to unmodified SS316L via an energy-dependent endocytosis mechanism. Altogether, these results highlight the potential of NT-SS316L as a promising implant material for localized drug delivery of different biomacromolecule cargoes and establish the serum protein corona profile on NT-SS316L and unmodified SS316L as useful data for understanding stainless steel implant function in future *in vivo* work.

## 2 Results and Discussion

**Figure 1:**
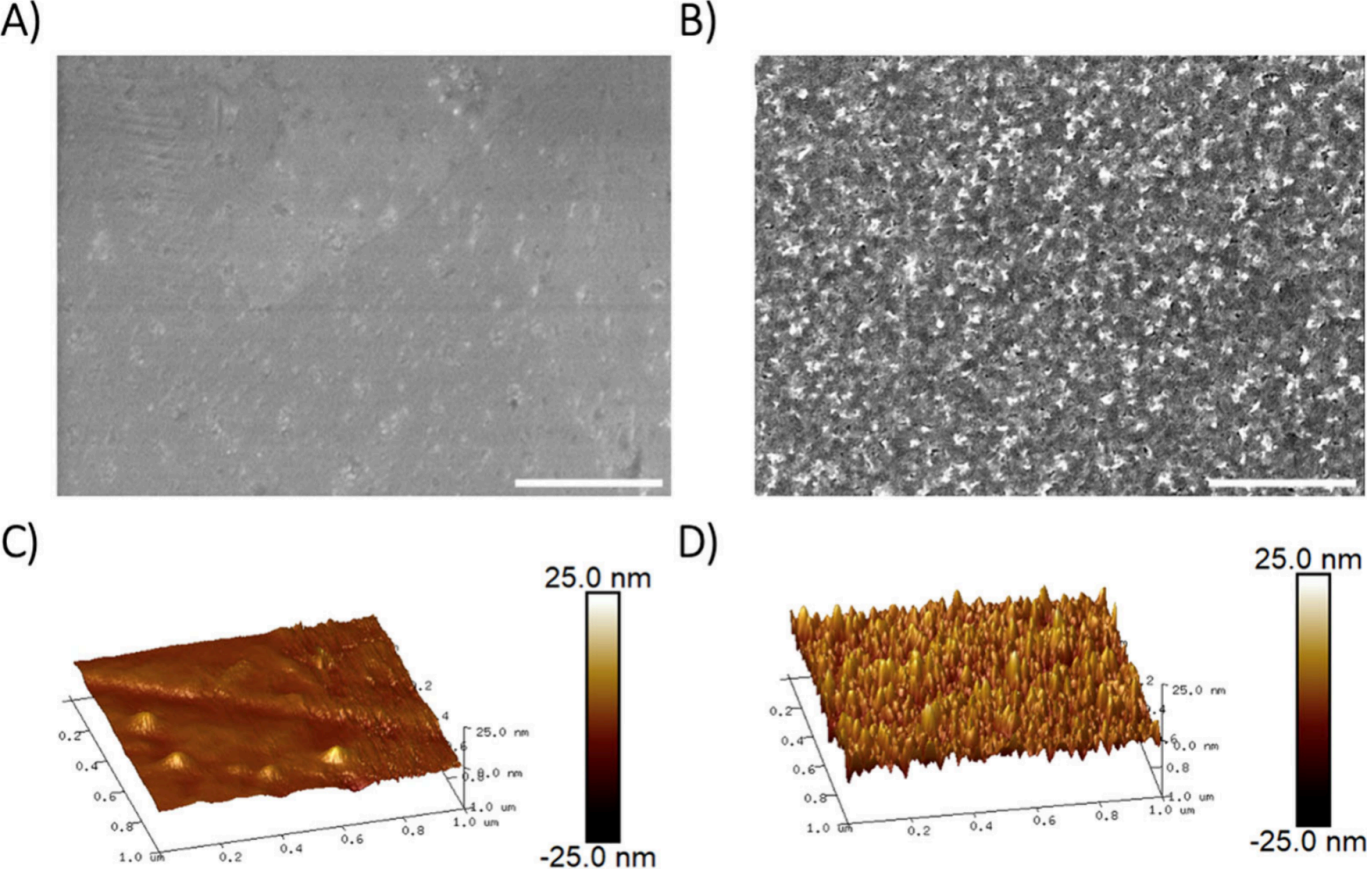
Physical characterization of nanotextured stainless steel. SEM images of SS316L (A) and NT-SS316L (B) nanoarchitecture, scale bars 500 nm. (*n* = 3) Atomic Force Microscopy images of SS316L (C) and NT-SS316L (D). (*n* = 3).

The nanofeatures on NT-SS316L were fabricated using a low-cost, industrially relevant electrochemical etching method similar to currently used electropolishing.^28,32,33^ In the etching process, SS316L samples are submerged into a solution of nitric acid, which acts as an electrolyte, and an electrical voltage is applied. This creates nanopores and sharp nanoprotrusions. Three-dimensional topographical features and morphologies were characterized by using scanning electron microscopy (SEM) and atomic force microscopy (AFM), with an unmodified stainless-steel sample serving as a control (Figure 1). The topographical features of NT-SS316L samples had nanoarchitecture that closely resembled our previous report.^28^ Arithmetic mean roughness (R_a_) and root mean squared (RMS) roughness (R_q_) were calculated from AFM images, and hydrophobicity was calculated using contact angle measurements. NT-SS316L displayed increased R_a_ values (5.93 ± 1.44 vs 1.60 ± 1.13) and R_q_ values (7.62 ± 2.41 vs 2.63 ± 1.40) compared to unmodified SS316L. SS316L and NT-SS316L surface height profiles showing the nanofeatures are shown in Figure S1. In addition, we observed a modest increase in hydrophobicity on NT-SS316L with a water contact angle of 93.7° ± 2.3° compared to 85.1° ± 2.0° for SS316L. Increased hydrophobicity is attributed to the high aspect ratio nanoarchitecture.^34^ The Cassie–Baxter wetting model suggests that liquid would not fully penetrate the nanopores on NT-SS316L, creating air pockets that alter contact angle measurements.^30^

**Figure 2:**
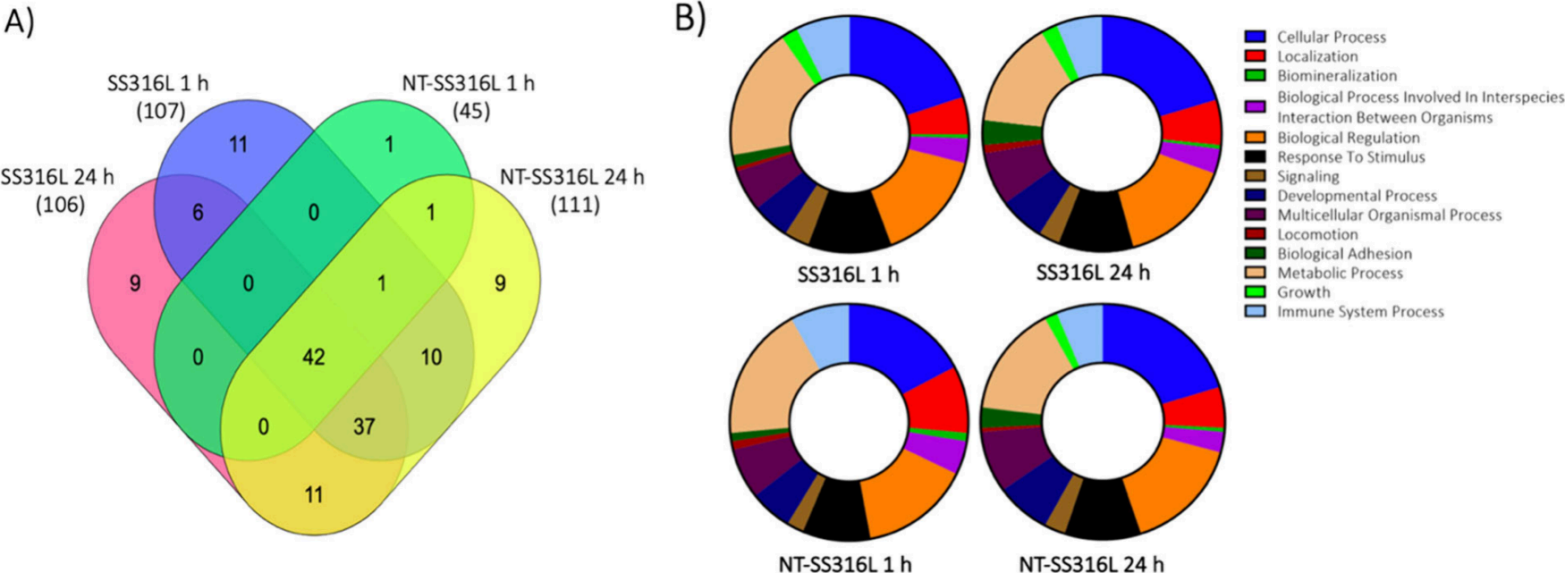
Protein corona composition and related biological function. (A) Venn diagram showing the overlapping identified FBS proteins on each steel surface after 1 and 24 h of incubation. The total number of identified proteins is listed in parentheses for each group. (B) Summary pie charts with biological function processes connected to each corona protein using PANTHER.

Metallic implants contact protein-rich biological fluids, such as plasma, upon their insertion into the body. Proteins in the fluids subsequently adsorb onto the implant surface, forming a layer of protein or a corona.^35−37^ This process, termed protein fouling, can facilitate microbial attachment and also increase interactions with cells from the innate immune system, such as macrophages, neutrophils, and monocytes.^38^ Protein fouling can cause inflammation or life-threatening infections, diminish implant lifetime, increase morbidity and mortality, and necessitate difficult and costly replacement surgery.^39,40^ While NT-SS316L is antibacterial, mammalian cells do adhere and characterization of adsorbed proteins and an understanding of adsorption kinetics is necessary to determine the biological fate of implant surfaces.^30,43−45^ We evaluated the formation of a hard protein corona on NT-SS316L and SS316L surfaces immersed in 10% fetal bovine serum (FBS) as a model for *in vivo* tissue conditions. Surfaces were incubated for 1 or 24 h at 37 °C and washed with multiple cycles of sterile water to remove excess and weakly associated proteins (soft corona). The number of necessary washing cycles was determined by measuring the amount of protein in the wash buffer using the bicinchoninic acid assay (BCA). (Figure S2A) The layer of strongly adsorbed proteins (hard corona) was removed from each surface by incubating with 2% sodium dodecyl (SDS) solution. The resulting protein solution was visualized by gel electrophoresis (SDS-PAGE) (Figure S2B). As expected from the corona literature, the mass of corona changed with time, decreasing by ∼half from 1 to 24 h.^41,42^ At both time points, we observed a 2-fold decrease of adsorbed protein density on NT-SS316L compared to SS316L (Figure S2C), which we attribute to steric hindrance from the NT-SS316L spike nanoarchitecture and reduced wettability deep in the pores. This result contrasts with that of nanotextured titanium alloys produced by hydrothermal treatment, which showed small increases in adsorbed proteins compared to untreated surfaces.^43−47^ Using shotgun proteomics, we detected 112 unique proteins with more than 10 peptide spectral matches in both NT-SS316L and SS316L coronas (XLSX Supplemental File). A list of the top 10 proteins adsorbed to each surface can be found in Table 1. Despite the differences in corona mass, the composition of the protein corona was highly conserved between NT-SS316L and SS316L at both time points (Figure 2A, Table 1). Primarily overlapping corona compositions were also reported in the nanotextured titanium alloy and control.^47^ Taken together, these results suggest that the chemical similarity of unmodified and nanotextured steel or titanium alloys dominates adsorbed protein identity, and the nanotexture exhibits more impact on corona mass than composition.

**Table 1:** Top 10 Identified Proteins in Each Protein Corona Determined Based on the Peptide Spectral Matches

	SS316L 1 h	Protein Corona Percentage (%)	SS316L 24 h	Protein Corona Percentage (%)	NT-SS316L 1 h	Protein Corona Percentage (%)	NT-SS316L 24 h	Protein Corona Percentage (%)
1	Serum albumin	26.6%	Serum albumin	23.5%	Serum albumin	21.0%	Serum albumin	18.6%
2	Hemoglobin fetal subunit beta	7.6%	Plasminogen	8.4%	Alpha-2-HS-glycoprotein	7.9%	Complement C3	6.9%
3	Complement C3	5.9%	Hemoglobin fetal subunit beta	7.9%	Keratin, type I cytoskeletal 10	7.7%	Serotransferrin	6.2%
4	Alpha-2-HS-glycoprotein	5.8%	Complement C3	7.2%	Serotransferrin	6.9%	Hemoglobin fetal subunit beta	5.2%
5	Plasminogen	4.5%	Hemoglobin subunit alpha	6.2%	Complement C3	6.7%	Plasminogen	4.6%
6	Hemoglobin subunit alpha	4.3%	Alpha-2-HS-glycoprotein	4.6%	Hemoglobin fetal subunit beta	5.7%	Alpha-2-HS-glycoprotein	4.1%
7	Serotransferrin	3.5%	Serotransferrin	3.1%	Plasminogen	5.3%	Keratin, type I cytoskeletal 10	3.7%
8	Keratin, type I cytoskeletal 10	1.8%	Keratin, type I cytoskeletal 10	1.7%	Keratin, type II cytoskeletal 1	5.2%	Hemoglobin subunit alpha	2.9%
9	Apolipoprotein A-I	1.8%	Complement factor H	1.5%	Hemoglobin subunit alpha	3.8%	Keratin, type II cytoskeletal 2 epidermal	2.8%
10	Alpha-fetoprotein	1.6%	Alpha-fetoprotein	1.4%	Keratin, type II cytoskeletal 2 epidermal	3.6%	Keratin, type II cytoskeletal 1	2.3%

The adsorbed protein corona alters the body’s response to implanted materials by changing their biological identity.^16^ Though the protein corona of nanoparticles has been well-studied, few reports have evaluated protein coronas on metallic implant materials, although existing data has demonstrated nanostructured surfaces’ increased ability to attract vitronectin and to reduce proinflammatory cytokine expression.^18,41^ Bioinformatics techniques have been widely used to understand the potential impact of protein corona on downstream biological functions.^31,42,46,47^ We used PANTHER (**p**rotein **a**nalysis **t**hrough **e**volutionary **r**elationships) to group individual proteins in the corona according to their biological functions (Figure 2B), using only proteins with more than 10 peptide spectra recorded by mass spectrometry. Proteins in the corona may be folded or unfolded, or in a state that is or is not able to engage with targets required for intended function, but the categorization of the protein corona can help understand the biological functional diversity of nanotextured material architecture. The most upregulated function from bioinformatics analysis was cellular processing (Figure 2B) which entails intracellular communication, cell adhesion, vesicle-mediated transport, and cell growth. Each of these processes have important implications for medical implants.^48,55^ The overall distribution of biological processes is relatively consistent across NT-SS316L and SS316L surfaces at both time points, as expected from the similar proteomics data.

Because PANTHER analysis does not account for the number of proteins located in the corona, we conducted fold change analysis to evaluate the top 5 up- and downregulated processes associated with the protein corona of NT-SS316 samples compared to SS316L samples (Table 2). We used g/Profiler, a tool for functional enrichment analysis gene ontology, to conduct this analysis (XLSX Supplemental File). It is important to note that identified processes are not actually occurring in our experiments, as no cells or organisms are present. Instead, g/Profiler analysis identifies potentially significant proteins from normalized percentages of spectral counts (NpSpCs), illumining possible downstream effects of interactions with the protein corona. Despite the coronas of unmodified and nanotextured samples being generally similar (Figure 2), g/Profiler analysis revealed an upregulation in wound healing processes (Table 2), which can be attributed to the high enrichment of proteins such as junction plakoglobin in the 24 h NT-SS316L protein corona measurement (XLSX Supplemental File). Junction plakoglobin, a cell–cell adhesion protein, promotes osteogenesis as a functional component of the Wnt signaling pathway and is vital for endothelial wound healing.^45,49^ The enrichment of junction plakoglobin in the NT-SS316L corona suggests that it could potentially contribute to bone regeneration if applied as an orthopedic implant material. Junction plakoglobin also mediates cell–cell adhesion as a part of the adherens junctions and desmosomes. Further, it interacts with molecules in the signal transduction pathway that regulates cell growth and morphogenesis.^49^ Adiponectin B demonstrated the same high fold change on NT-SS316L at 24 h as junction plakoglobin (XLSX Supplemental File). An adipokine, adiponectin B inhibits expression of proinflammatory cytokines and adhesion molecules, reducing excessive immune response, and promotes angiogenesis.^50,51^ Dihydrolipoamide S-succinyltransferase (DLST) was also enriched on the NT-SS316L protein corona at the 24 h time point (XLSX Supplemental File). DLST is critical for energy production in the tricarboxylic acid cycle, which promotes cell growth and healing. Inhibition of DLST has been shown to reduce ATP production by over 40%, interrupting energy production and associated cellular processes.^52^ Thus, enriched adsorption of specific corona proteins, like those discussed here, could suggest a potential for improved healing and integration of nanotextured steel, which can be directly assessed in future work. The ability of nanostructured stainless steel to support a hard corona also motivates manipulating the corona for delivery of functional cargoes.

**Table 2:** Top 5 Upregulated and Downregulated Biological Functions from NT-SS316L Protein Corona Compared to Unmodified SS316L^a^

**1 h**		**24 h**	
*Upreg.*^b^	*Downreg.*^c^	*Upreg.*^b^	*Downreg.*^c^
Cytoskeletal Protein-Membrane Anchor Activity	Membrane Attack Complex	Wound Healing	Structural Constituent Of Cytoskeleton
Intermediate Filament	Humoral Immune Response	Collagen Trimer	
Gamma-Catenin-Tcf7L2Complex	Proteolysis	Cell Adhesion	
	Blood Coagulation	Extracellular Matrix	
	Extracellular Region	Extracellular Region	

a Results were generated by g/Profiler functional enrichment analysis.

b Upregulated biological function from g/Profiler from pooled two MS runs per each independent sample.

c Downregulated biological function from g/Profiler from pooled two MS runs per each independent sample.

To test the ability of the NT-SS316L hard corona for intracellular delivery of biomacromolecules, we evaluated two charged variants of superfolder green fluorescent protein (sfGFP) with (−10) and (+10) net surface charges as model cargoes (Figure S3). We adsorbed 1 mg/mL of each variant onto a confined surface area of NT-SS316L or SS316L to form an sfGFP protein corona over 24 h. Samples were washed with water to remove weakly adsorbed protein (Figure S4), leaving the sfGFP hard corona. There was no major difference in adsorption levels with 21% and 20% of incubated sfGFP(−10) adsorbed on NT-SS316L and SS316L, respectively, and 20% and 15% of incubated sfGFP(+10) adsorbed on NT-SS316L and SS316L, respectively. The NT-SS316L was capable of generating a corona from a single source, as has been reported for unmodified steel alloys with bovine serum albumin or lysozyme.^18^ NT-SS316L was visualized with scanning electron microscopy after protein loading to ensure that the characteristic nanotexture topography was maintained (Figure S5).

**Figure 3:**
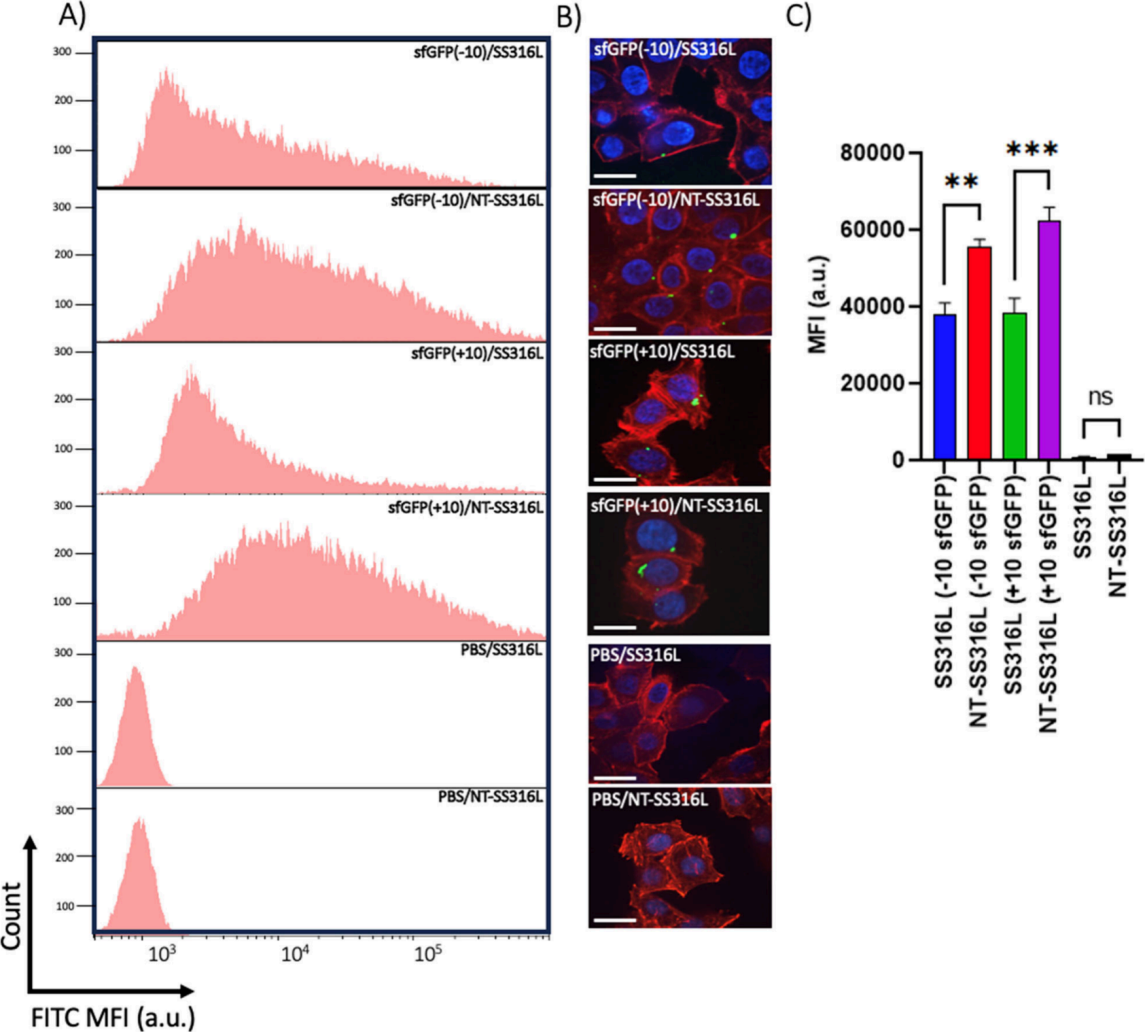
NT-SS316L or SS316L mediated internalization of sfGFP variant internalization by HeLa cells measured by flow cytometry and confocal microscopy. (A) Representative flow cytometric profiles of HeLa uptake after 24 h incubation on sfGFP coated surfaces at 37 °C. (B) Representative confocal images (*n* = 10–15) of sfGFP internalization. (Scale bars −30 μm; sfGFP variant–green, actin cytoskeleton–red, nuclei–blue). (C) Summary of flow cytometry data, Median Fluoresce Intensity: MFI (*n* = 3); (one-way ANOVA with Tukey’s post hoc analysis, **p < 0.01, ***p < 0.001)

Intracellular delivery efficacy of sfGFP (−10) and (+10) was assessed in HeLa cells as a model cell line following 24 h of incubation of cells on surfaces in FBS supplemented media using flow cytometry and fluorescence microscopy (Figure 3A,B). Trypan blue was used to quench extracellular sfGFP fluorescence. NT-SS316L demonstrated significantly increased sfGFP delivery efficacy for both charge variants compared to unmodified SS316L controls, with an ∼55% increase in uptake for sfGFP (+10) and ∼30% increase for sfGFP (−10) (Figure 3C). The kinetics of protein desorption of sfGFP on SS316L and NT-SS316L were also analyzed. sfGFP coated surfaces were incubated with FBS for 48 h and subsequent sfGFP release was measured and correlated with a sfGFP-FBS calibration curve. Only less than 2% of sfGFP desorbed from NT-SS316L and SS316L (Figure S6), indicating that interfacial uptake, not endocytosis of free-floating protein, is the mechanism of protein delivery from steel surfaces. As both positive and negative sfGFP were delivered, the lack of strong dependence on protein surface charge on surface adsorption and cellular uptake suggests that NT-SS316L could be applicable to a wide range of proteins.

**Figure 4:**
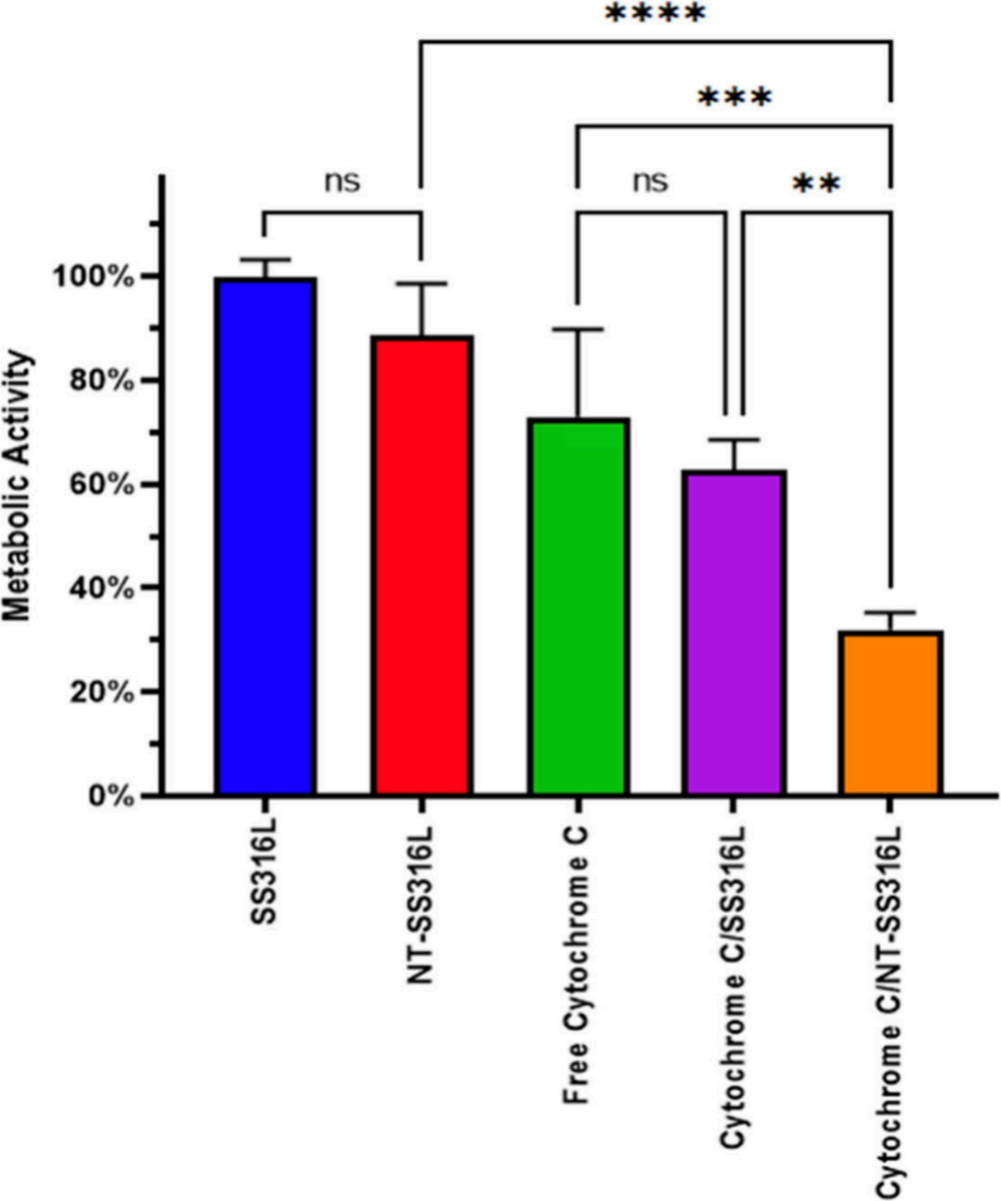
HeLa cytotoxicity of SS316L, NT-SS316L, free cytochrome c, cytochrome c coated onto SS316L, and cytochrome c coated onto NT-SS316L after 24 h incubation at 37 °C. HeLa cells on polystyrene tissue culture plate was used as reference for normalization. (one-way ANOVA with Tukey’s post hoc analysis, **p < 0.01, ***p < 0.001, ****p < 0.001).

To evaluate whether NT-SS316L could deliver functionally active proteins to the cytosol, we assessed cytochrome c delivery. Cytochrome c activates apoptotic signaling by engaging with protease activating factor-1 (APAF1). The resulting complex forms an apoptosome to activate caspase-9, leading to apotosis.^53^ This downstream cascade can only occur once functional cytochrome c is exposed to the cytosolic environment and has previously been used as a model cargo to induce apotosis.^54−56^ To measure functional cytosolic delivery, we generated a cytochrome c hard corona on SS316L and NT-SS316L samples and exposed them to HeLa cells for 24 h. MTT assay demonstrated that nanotextured steel delivered cytochrome c more effectively than SS316L, evidenced by a significantly larger reduction in metabolic activity (Figure 4). Soluble and SS316L adsorbed cytochrome c demonstrated similar reductions in metabolic activity (∼30% and ∼35%), while NT-SS316L demonstrated a 2-fold higher reduction in metabolic activity (∼68%). Additionally, the PBS treatment groups (no adsorbed cytochrome c) indicated that adhesion to NT-SS316L did not induce metabolic changes in HeLa cells compared to SS316L.

**Figure 5:**
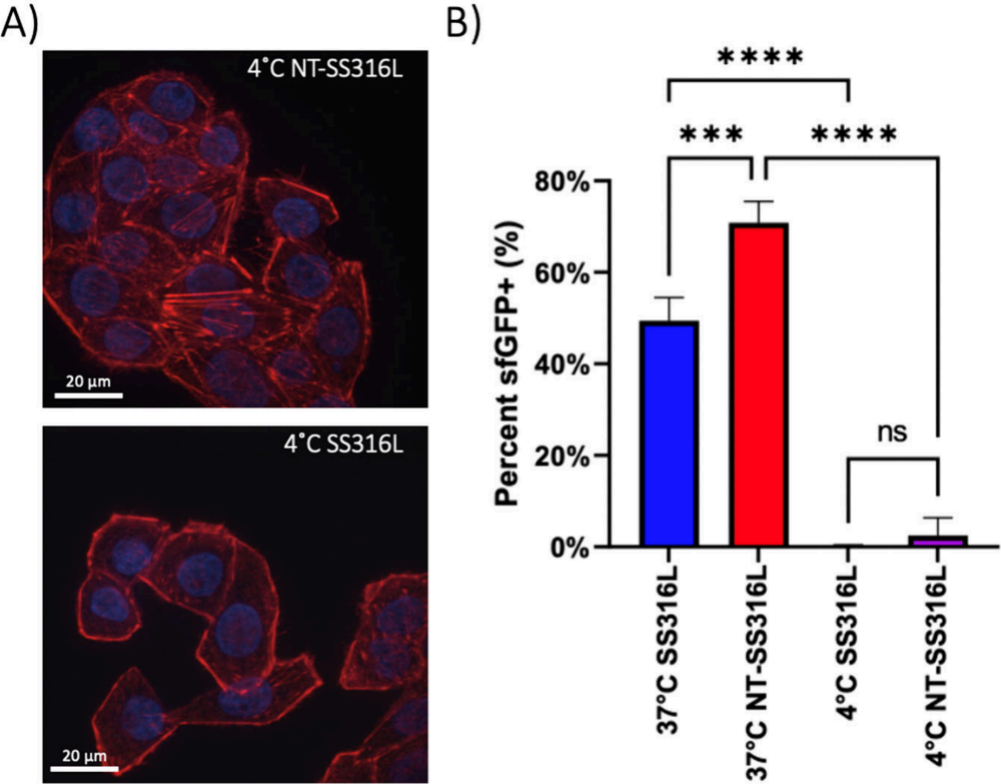
Endocytosis of biomacromolecule inhibition mediated by temperature on NT-SS316L and SS316L for HeLa Cells. (A) Representative confocal images (*n* = 10–15) adhered cells on either NT-SS316L or SS316L when incubated at 4 °C to inhibit endocytosis. (Scale bar −30 μm; sfGFP variant–green, actin cytoskeleton–red, nuclei–blue). (B) Summary of flow cytometry data indicating% of cells that are GFP positive (*n* = 3–4); (one-way ANOVA with Tukey’s post hoc analysis, ***p < 0.001, ****p < 0.001).

Some nanostructures on surfaces can be engulfed directly by cells or stimulate endocytosis of surface-bound cargo.^57^ Nanoarchitecture features have been noted to greatly improve the processing of molecules in the extracellular matrix. Exposure to gold nanospike arrays locks cells in the G2 phase, where improved delivery of various soluble biomacromolecules is observed.^20^ Nanospike exposure may even promote uptake of biological molecules through dynamic interactions with the cell membrane.^58−62^ To understand the mechanism of uptake, either through penetration of the membrane by nanoarchitecture or enhanced endocytosis, we evaluated internalization at 4 °C, which inhibits energy-dependent endocytic uptake.^57,63^ HeLa cells were adhered onto sfGFP-enriched NT-SS316L or SS316L surfaces under standard culture conditions (37 °C) for 2 h, and the temperature was subsequently lowered for 24 h. Flow cytometry analysis detected a significant decrease in GFP positive cells at low temperature, suggesting that both NT-SS316L and SS316L uptake is endocytosis-mediated (Figure 5). However, more studies are needed to observe the increased formation of endocytic vesicles at the cell-nanotextured steel interface.^64^

**Figure 6:**
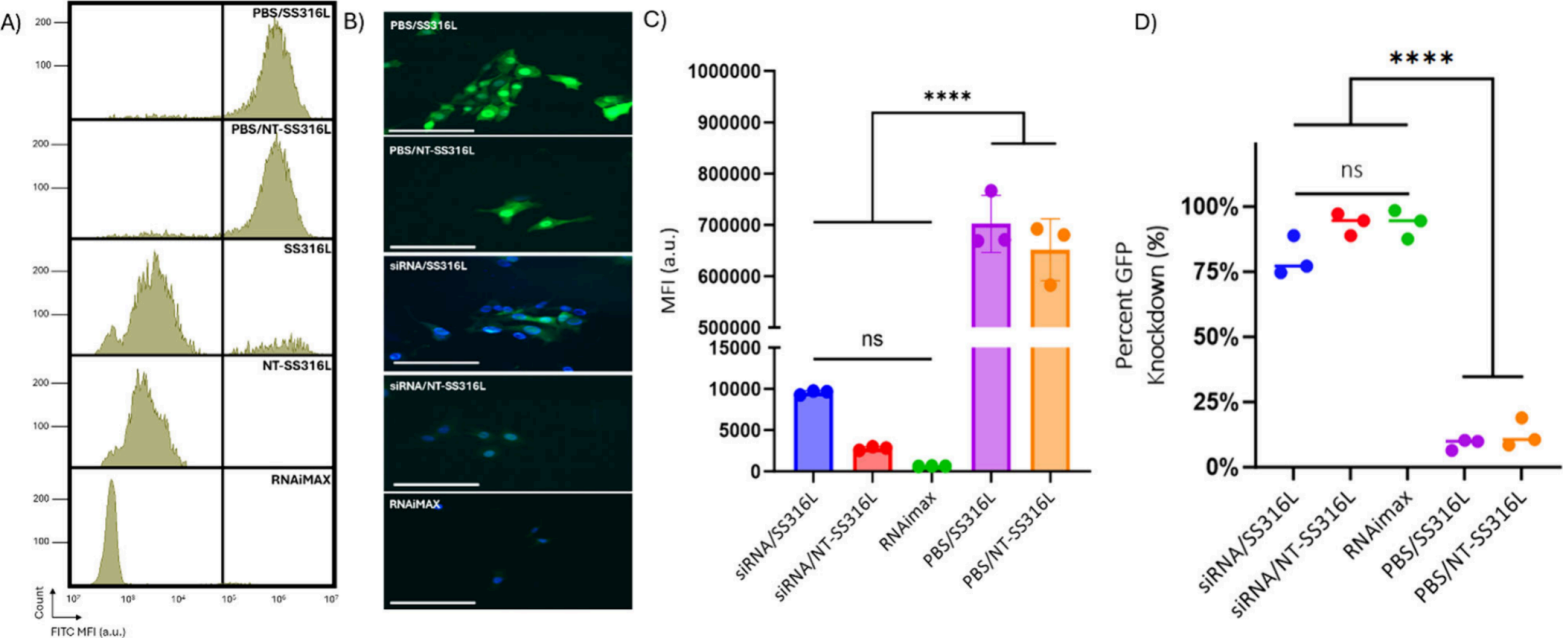
GFP knockdown in NIH3T3 GFP expressing fibroblasts cells measured by flow cytometry and confocal microscopy mediated through GFP silencer siRNA coated on NT-SS316L or SS316L. (A) Representative flow cytometric profiles of NIH3T3 GFP cells on NT-SS316L or SS316L surfaces after 48 h. B) Representative confocal images (*n* = 7) of GFP knockdown. (Scale bars −120 μm; GFP expression–green, nuclei–blue). C) Summary of flow cytometry data, MFI and percent knockdown (*n* = 3); (one-way ANOVA with Tukey’s post hoc analysis ****p < 0.0001)

In addition to protein delivery, we assessed the delivery of short interfering RNA (siRNA) by NT-SS316L. siRNA delivery is of specific interest to combat gene overexpression by post-transcriptional regulation.^65^ Implant surgery and materials often increase levels of pro-inflammatory cytokines such as interleukin-6, interleukin-8, and tumor necrosis factor (TNFα) that can enable persistent inflammatory responses, preventing healing and integration, leading to bone degradation and loosing of implants.^66^ Therefore, modulating levels of pro-inflammatory cytokines at implant sites is a promising strategy to overcome these challenges. Typically, delivery of siRNA requires a carrier, such as lipid nanoparticles. Systemic siRNA delivery is an unfavorable alternative to local delivery, because it may lead to undesirable side effects. Implant-based siRNA delivery has been successful using dopamine-coated SS316L, and even unmodified SS316L coated with siRNA was stable enough to achieve knockdown of firefly luciferase (albeit at a lower percentage than dopamine-coated) *in vitro*.^9^ “Naked” siRNA has also been successfully delivered using siRNA-coated steel microneedles.^67^ To investigate functional siRNA delivery, we measured the knockdown of GFP in NIH3T3 GFP-expressing fibroblast cells on NT-SS316L coated with Silencer GFP siRNA using flow cytometry and confocal microscopy (Figure 6). Consistent with the described siRNA coated steel literature, we observed knockdown on both NT-SS316L and SS316L, comparable to the RNAiMax Lipofectamine control.

## 3 Conclusion

Demand for metallic implants for orthopedic and dental surgery is at an all-time high, reflecting millions of procedures each year and a projected cost of more than $1.62 billion.^3^ Inhibited surgical wound healing and implant integration present a major problem for patients, leaving them vulnerable to bacterial infections that can be life-threatening and require repeat surgery. We synthesized nanotextured stainless steel using electrochemical etching to manipulate the surface interaction between stainless steel and cells. Our analysis found that a stable hard protein corona is generated on both NT-SS316L and unmodified steel, and this strong adsorption could be exploited for intracellular biomacromolecule delivery. We determined that NT-SS316L promotes energy-dependent uptake, increasing the intracellular delivery of three model proteins and siRNA to HeLa and NIH3T3 fibroblast cells. Overall, NT-SS316L is a promising antibacterial implant material and potential platform for delivery of wound healing or anti-inflammatory biotherapeutics to implant sites. This proof-of-concept study provides an opportunity for future work to explore the delivery of proteins that enable other cellular functionalities, such as proliferation and intracellular signaling. Future studies will evaluate implant applications of NT-SS316L coated with therapeutic biomacromolecules *in vivo* to determine wound healing and antibacterial efficacy.

## 4 Materials and Methods

### 4.1 Nanotextured Steel Sample Preparation

SS316L plates (2.54 × 1.27 × 0.2 cm, Maudlin Products) were cut using a water jet cutter. One SS316L sample was used as the counter electrode, while the plates to be etched were used as the working electrodes. Prior to anodization, samples were vigorously rinsed with acetone (ACS reagent, ≥99.5%, Sigma-Aldrich), methanol (ACS reagent, ≥99.5%, Sigma-Aldrich), 2-propanol (ACS reagent, ≥99.5%, Sigma-Aldrich), and deionized water to remove organic contaminants. Caution: incomplete removal of organic molecules can lead to dangerous chemical reactions with nitric acid.^68^ Samples were subsequently air-dried at ambient temperature, and SS316L plates were partially masked with insulating vinyl medium adhesion cleanroom tape (3M Science) to protect the working electrode from corrosion. An active working area of (∼1.61 cm^2^) was maintained for anodization. Stainless-steel wires connected both the working and counter electrodes to the function generator (N7970A Advanced Power System, Dynamic DC Power Supply, 9 V, 200 A, 2000 W). The counter electrodes were placed in a customized glass container and immersed in nitric acid solution (48 wt %, Sigma-Aldrich), which served as the electrolyte. A potentiostatic polarization was applied to the system at 8 V for 30 s at room temperature, which generated nanotextured NT-SS316L surfaces. After anodization, NT-SS316L plates were washed extensively with deionized water and dried at room temperature for 24 h before surface characterization.^28^

### 4.2 Surface Characterization

NT-SS316L and SS316L samples were cleaned using a Branson CPX8800 Digital Ultrasonic Cleaner for 20 min in acetone, isopropyl alcohol, and finally washed with DI water. Samples were autoclaved and then air-dried overnight at room temperature under sterile conditions. Surface morphologies of NT-SS316L and SS316L samples were characterized using SEM (Hitachi SEM SU8010) at 5 kV acceleration potential. Topographical information was acquired by AFM (Bruker ICON AFM) using an antimony (n)-doped Si AFM probe (TESPA-V2, Bruker). The surface roughness parameters of samples were obtained by AFM measurements from scanning a surface area of 1 μm^2^ while avoiding artificial defect areas using NanoScope Analysis v1.40 (Bruker). Surface hydrophobicity measurements were made by using a goniometer (RaméHart-290). Distilled water droplets (4 μL) were dispensed on samples, and the contact angle was analyzed with RaméHart software.

### 4.3 Proteomic Analysis on NT-SS316L and SS316L

SS316L samples with or without nanotexture were incubated in 3 mL of 10% fetal bovine serum (FBS, Thermo Fisher Scientific) for 1 and 24 h at 37 °C. Vinyl tapes (3M Science) were used to cover the nontextured area and unbound proteins were washed away 3 times by rocking samples with 1 mL of Milli-Q water (18.2 MΩ·cm) for 10 min at room temperature. After each washing cycle, the wash solutions were collected and stored for further analysis. After the last wash cycle, samples were resuspended in 200 μL of 2% SDS and incubated for 10 min at room temperature. After incubation, 800 μL of Milli-Q water was added, and the samples were incubated for an additional 1 h at room temperature while rocking. After incubation, the solution was carefully extracted and concentrated to 100 μL using a Proteomic Centrivap Concentrator System (Labconco). The concentrations of the resulting protein sample solution and wash supernatants were measured using BCA assay. SDS-PAGE was used to visualize the proteins in each sample solution. 7 μg of protein was mixed with 7 μL of Laemmeli buffer solution (Sigma-Aldrich) and incubated for 5 min at 95 °C. The resulting solutions and a Spectra Multicolor Broad Range Protein Ladder (Thermo Fisher Scientific) were separated with a 4–20% mini-PROTEAN TGX Precast protein gel (Bio-Rad Laboratories) at 150 V for 40 min. Proteins were stained with Coomassie Blue and imaged using a Gel Dox XR+ Gel Documentation System. Molecular weight analysis and densiometry were performed on the gel to measure the intensity of each identified molecular weight band using a Fiji instrument (ImageJ). Protein sample solutions (n = 3) were reduced, alkylated, and digested with trypsin according to the FASP protocol.^69^ The peptides were analyzed by nano-LC-MS/MS (liquid chromatography–MS Q Exactive Plus Hybrid Quadrupole-Orbitrap with Thermo UltiMate 3000 HPLC System), and peptide identification was performed as previously described with the following modifications.^70^ Reverse phase chromatography was performed using an in-house packed column (40 cm long × 75 μm ID × 360 μm OD, Dr. Maisch GmbH ReproSil-Pur 120 C18-AQ 1.9 μm beads) and a 120 min gradient. The raw files were searched using the Mascot algorithm (ver. 2.5.1) against a protein database constructed by combining the sequences of the Uniprot Bovine reference database (downloaded 5–22–19, 20,303 entries) and a contaminant database (cRAP, downloaded 11–21–16 from http://www.thegpm.org) via Proteome Discoverer 2.1. Only peptide spectral matches with an expectation value of less than 0.01 (“High Confidence”) were used. Protein molecular weight (MW) was obtained from UNIPROT. The spectral count of each identified protein was normalized by MW to determine the relative quantitative concentrations of each protein in the protein corona of each NP by using eq 1. Percentage corona coverage of the surfaces was calculated using the PSM abundancy and normalized for their relative values (XLSX Supplemental File).
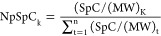
1

NpSpCk is the normalized percentage of the peptide spectral count for each protein k, SpC is the peptide spectral count identified using nano- LC–MS/MS, MW is the molecular weight (Da) of protein k, and n is the total number of proteins identified.

### 4.4 Production of sfGFP Variant Proteins

The pET28a plasmids containing the genes for sfGFP (+10) and (−10) variants with 6x-his tags (GenScript Biotech) were transformed and expressed in *Escherichia coli* strain BL21*(DE3). To express the proteins, 1 L of lysogeny broth (LB, Sigma-Aldrich) containing 50 mg of kanamycin (Sigma-Aldrich) was inoculated with 5 mL of overnight culture at 37 °C, and expression was induced with 1.0 mM isopropyl β-D-1 thiogalactopyranoside (Sigma-Aldrich) once the culture reached an optical density at 600 nm of 0.6–0.8. Then, 5 h after induction, cultures were harvested by centrifugation at 4000xg for 15 min. The pellets were resuspended in lysis buffer containing 300 mM NaCl, 50 mM NaH_2_PO_4_, and 10 mM imidazole (Sigma-Aldrich) and lysed by sonication. After centrifugation at 10000xg for 30 min, the supernatant was incubated with Ni-nitrilotriacetic acid agarose resin (Qiagen) for 1 h at 4 °C. The suspension was flowed through an Econo-Column (Bio-Rad laboratories) and washed with 100 mL of lysis buffer containing 25 mM imidazole. 10 mL elutions were collected using lysis buffer with 250 mM imidazole. After verifying the purity by SDS-PAGE using SeeBlue Plus2 Prestained Protein Standard (Thermo Fisher Scientific) as ladder reference, the purified proteins were buffer exchanged into phosphate buffered saline (PBS) by dialysis with a 3.5 kDa cutoff membrane (Cole-Parmer) with four buffer exchanges at 4 °C over 24 h. Protein concentrations were determined by using a BCA assay.

### 4.5 Biomacromolecule Surface Adsorption

NT-SS316L and SS316L samples were sterilized by autoclave and then incubated with 100 μL of sfGFP variants (1 mg/mL) or cytochrome c for 24 h at 4 °C in mammalian cell cloning PYREX rings (Corning) attached to the steel surfaces with clear nail polish (OPI). Unbound proteins were washed away using deionized water, samples were washed two times, and supernatants were collected for the BCA assay of protein concentration in each wash. For siRNA loading, sterilized samples were washed with RNase AWAY (Thermo Fisher Scientific) overnight and then dried. Mammalian cell cloning rings were added onto samples, and 100 μL of 300 nM of Silencer GFP (eGFP) siRNA (Thermo Fisher Scientific) diluted with DNase/RNase-Free distilled water was added and incubated on surfaces for 24 h at 4 °C. Samples were gently washed with DNase/RNase-Free distilled water (Thermo Fisher Scientific).

### 4.6 Cell Culture

HeLa cells (American Type Culture Collection; taken from Henrietta Lacks, http://henriettalacksfoundation.org/) were cultured in Dulbecco’s modified Eagle’s medium (DMEM) (Thermo Fisher Scientific), supplemented with 10% (v/v) premium fetal bovine serum (FBS) and 1% (v/v) penicillin/streptomycin (Thermo Fisher Scientific). Passages 5–15 were used in the described experiments. NIH3T3/GFP cells (MyBioSource) were cultured in DMEM (high glucose) supplemented with 10% (v/v) FBS, 0.1 mM MEM nonessential amino acids, 2 mM l-glutamine, 1% (v/v) penicillin/streptomycin, and 10 ug/mL Blasticidin (Thermo Fisher Scientific). Passages 3–10 were used in the described experiments. All cells were incubated under standard conditions (Humid, 5% CO_2_ atmosphere, 37 °C).

### 4.7 Flow Cytometry

HeLa cells were incubated at 4 × 10^4^ cells per cloning ring on steel surfaces with adsorbed proteins in triplicate for 24 h in supplemented media at 37 °C. For the 4 °C endocytosis inhibition study, HeLa cells were added onto sfGFP (−10) coated surfaces for 2 h in supplemented media at 37 °C to enable attachment and then placed at 4 °C for 24 h. Cells were washed twice with PBS, briefly trypsinized using trypsin - ethylenediaminetetraacetic acid (EDTA) (0.25%, phenol red), then filtered with 40 μm cell strainers (Fisher Scientific) and resuspended in chilled media with 0.4% trypan blue (Thermo Fisher Scientific) to quench extracellular green fluorescence. Gating was established using cells on surfaces without adsorbed sfGFP. Cell fluorescence was measured using a Beckman Coulter CytoFLEX.

NIH3T3/GFP cells were incubated at 2 × 10^4^ cells per cloning ring in triplicate in supplemented media at 37 °C for 48 h on steel with adsorbed siRNA. Cells were washed twice with PBS, briefly trypsinized, and then filtered with 40 μm cell strainers. Gating was established using untreated negative control cells and cells transfected with Lipofectamine RNAiMAX (Thermo Fisher Scientific) with Silencer GFP (eGFP) siRNA (Thermo Fisher Scientific) following manufacturer’s instructions. Cell fluorescence was measured using a Beckman Coulter CytoFLEX.

### 4.8 Cell Imaging

HeLa cells in supplemented media were seeded at a density of 4 × 1 × 10^4^ cells in cloning rings attached to surfaces with adsorbed sfGFP variants. After 24 h at 37 °C, cells were washed three times with ice cold PBS and fixed with 3.7% (v/v) paraformaldehyde (Fisher Scientific) for 10 min at room temperature followed by three washes with ice cold PBS. Cells were incubated with 0.0528 μM rhodamine phalloidin (Biotium) in blocking buffer, 1% BSA fraction V (Thermo Fisher Scientific) in PBS supplemented with 0.1% (v/v) Triton X-100 (Fisher Scientific) for 15 min at room temperature to stain actin. Cloning rings were removed and steel immobilized onto a glass slide. Cell nuclei were stained with antifade mounting medium containing DAPI (Vector Laboratories) and covered using #1.5 cover glass. Slides were imaged using an inverted PerkinElmer UltraVIEW VoX spinning disk confocal microscope. NIH3T3/GFP in supplemented media were seeded at a density of 2 × 10^4^ cells per well in cloning rings on surfaces coated with Silencer GFP (eGFP) siRNA. After 48 h at 37 °C, cells were processed the same as for HeLa cells.

### 4.9 Intracellular Delivery of Cytochrome C

Cytochrome c (>95%, Equine Heart, Lee Biosolutions Inc.) was adsorbed on surfaces as described above and cloning rings attached. HeLa cells in 10% FBS supplemented medium were seeded at a density of 1 × 10^4^ cells and incubated on the surfaces for 24 h at 37 °C. Cell metabolic activity was determined using a quantitative colorimetric conversion assay of 3-[4,5-dimethylthiazol-2-yl]-2,5-diphenyl tetrazolium bromide (MTT, Biotium). Following the manufacturer’s specifications, MTT solution was added, and cells were incubated for 4 h at 37 °C. The formazan product was solubilized by adding dimethylsulfoxide (DMSO, Thermo Fisher Scientific) to each well and mixed by pipet. The reacted solution was transferred to a well plate; absorbance was measured using a Synergy 2 Microplate reader (Bio-TEK Instruments) at 570 nm; and the background absorbance was measured at 630 nm. Cytocompatibility was compared between samples by normalization of the average cell metabolic activity of cells in control wells to 100%.

### 4.10 Statistical Analysis

Statistical significance was determined using a two-tailed Student’s *t* test in comparing two different conditions. One-way ANOVA was used to analyze the significant difference among 3 or more groups. P values less than 0.05 were significant (*, p < 0.05; **, p < 0.001; ***, p < 0.005, ****p < 0.0001) were determined by the posthoc test listed. The analysis was performed with GraphPad Prism (version 9.01).

## Supporting Information Available

Atomic force microscopy and scanning electron microscopy characterization, protein corona characterization using SDS-PAGE, sfGFP protein sequences, purification and zeta potential, soft corona washing of sfGFP protein on steel surface, sfGFP protein corona desorption kinetics in FBS, additional methods (PDF)
Protein sequence and list of the identified proteins using LC-MS/MS with NpSpC, PSM count, Fold Change, and a complete list of biological pathways by G:Profiler (XLSX)

## Abbreviations

AFM: Atomic Force Microscopy
APAF1: Activating Factor-1
ANOVA: Analysis of Variance
BCA: Bicinchoninic Assay
BSA: Bovine Serum Albumin
DAPI: 4′,6-Diamidino-2-phenylindole
DI Water: Deionized Water
DLST: Dihydrolipoamide S-Succinyltransferase
DMEM: Dulbecco’s Modified Eagle’s Medium
DMSO: Dimethylsulfoxide
EDTA: Ethylenediaminetetraacetic acid
FBS: Fetal Bovine Serum
IgG1: Immunoglobulin G1
LC–MS/MS: Liquid Chromatography–Tandem Mass Spectrometry (LC–MS/MS)
MW: Molecular Weight
MTT: 3-[4,5-Dimethylthiazol-2-yl]-2,5-diphenyl tetrazolium bromide
NT-SS316: Nanotextured Stainless Steel 316
NpSpCs: Normalized Percentages of Spectral Counts
PANTHER: Protein Analysis Through Evolutionary Relationships
PBS: Phosphate-Buffered Saline
RMS: Root Mean Squared
R_a_: Arithmetic Mean Roughness
R_q_: Roughness
SDS: Sodium Dodecyl Sulfate
SDS-PAGE: Sodium Dodecyl Sulfate–Polyacrylamide Gel Electrophoresis
SEM: Scanning Electron Microscopy
SS316L: Stainless Steel 316
SpC: Peptide Spectral Count
TNFα: Tumor Necrosis Factor
sfGFP: Superfolder Green Fluorescent Protein
siRNA: Short Interfering RNA
