# Supplementary material for: Nanoetched Stainless Steel Architecture Enhances Cell Uptake of Biomacromolecules and Alters Protein Corona Abundancy
Source: ACS Appl Mater Interfaces. 2024 Oct 17;16(43):58427–38. doi: 10.1021/acsami.4c14492 (PMC11533172; doi:10.1021/acsami.4c14492)
Supplement: Supplementary file 1 — am4c14492_si_001.pdf [file am4c14492_si_001.pdf]

Supporting Information

Nanoetched Stainless Steel Architecture Enhances Cell Uptake of Biomacromolecules and Alters Protein Corona Abundancy

Thomas Pho[a, b], Maeve A. Janecka [a], Samantha M. Pustulka [a], Julie A. Champion[a, b*]

School of Chemical & Biomolecular Engineering, Georgia Institute of Technology,

950 Atlantic Drive NW, Atlanta, Georgia 30332, United States

*Phone: 404-894-2874. E-mail: julie.champion@chbe.gatech.edu

Supplemental Methods

Protein Characterization and Zeta Potential

Protein concentrations were determined using a BCA assay following the manufacturer's protocol (Thermo Fisher Scientific). Zeta potential was assessed by electrophoretic light scattering in 10% PBS, respectively, with a Malvern Zetasizer Nano ZS90 (Malvern Instruments). Measurements were carried out in triplicate with three distinct samples. Each measurement consisted of 12–30 runs. Electrophoretic mobility was converted to zeta potential using the Smoluchowski approximation.

Adsorption kinetics and characterization of hard corona sfGFP in FBS media

NT-SS316L and SS316L were coated with 0.10 mg of sfGFP (-10) overnight at 4˚C. Surfaces were extensivity washed three times with Milli-Q water and 100 uL fetal bovine serum (FBS, Gibco) was added. Samples were placed in standard conditions (Humid, 5% CO_2 atmosphere, 37 ˚C) and supernatants of individual samples were measured at 485/510 nm (ex/em) in a flat black 96 well plate (corning) at varying timepoints. Signals were correlated using a calibration curve of known sfGFP amount in FBS. For characterization of the hard corona, samples had their supernatant removed and were washed once with Milli-Q water (18.2 MΩ·cm). Laemmli SDS sample buffer (Thermo Fisher Scientific) was added and incubated with steel samples for 20 min to release protein from surface. Samples were then boiled at 95˚C for 5 mins and analyzed by SDS-PAGE Mini-PROTEAN® Precast Gels using .

Evaluation of surface morphology after hard protein corona coating

Nanotextured steel samples were cleaned using a Branson CPX8800 Digital Ultrasonic Cleaner for 20 minutes in acetone (ACS reagent, ≥ 99.5%, Sigma-Aldrich), followed by isopropyl alcohol (ACS reagent, ≥ 99.5%, Sigma-Aldrich) and finally washed with DI water. Samples were autoclaved and then airdried overnight at room temperature under sterile conditions. sfGFP (-10) variant was coated onto NT-SS316L plates overnight at 4˚C for 24 h and then washed extensively. Protein on NT-SS316L was dehydrated using increasing concentrations of ethanol in DI water (10, 20, 40, 60, 70, 90, 100% (v/v)) with 10 min intervals between steps. Surfaces were treated with hexamethyldisilazane (reagent grade, 99%, Sigma-Aldrich) overnight under a flume hood. Surface morphology of NT-SS316L samples were characterized using scanning electron microscopy (SEM, Hitachi SEM SU8010) at 3.5 kV acceleration potential.

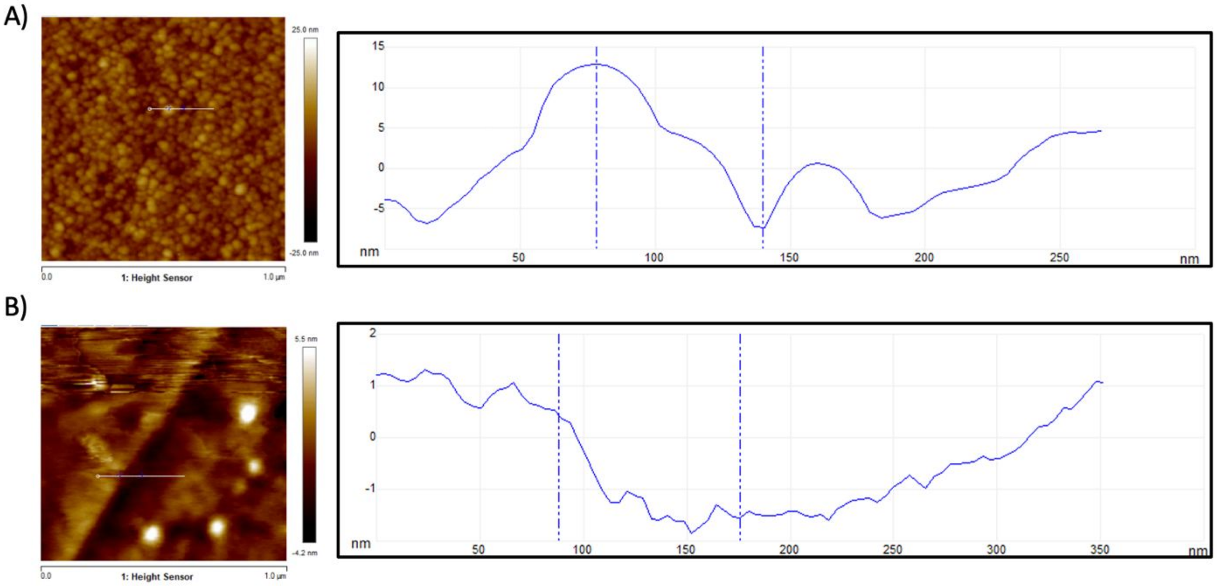

Figure S1. Surface height profiles of A) NT-SS316L and B)SS316L obtained by AFM.

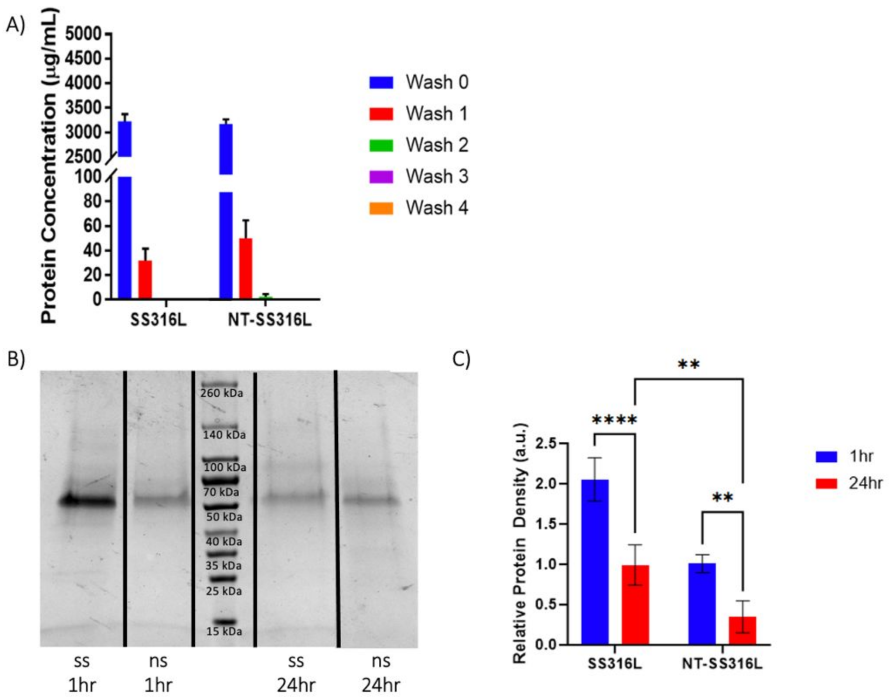

Figure S2. A) BCA assay measurement for soft corona removal from SS316L and NT-SS316L surface from FBS corona. B) Hard corona from SS316L (SS) and NT-SS316L (NS) at 1 h and 24 h timepoint with Spectra Multicolor Broad Range Protein Ladder (Thermofisher) (C) Densitometry from SDS-PAGE gel of the hard corona at 1 h and 24 h.

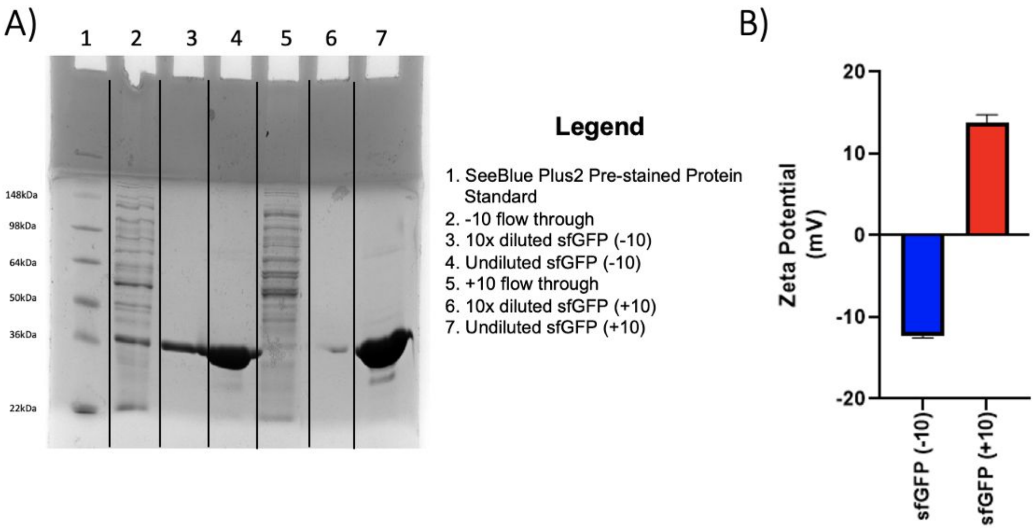

Figure S3. A) SDS-PAGE of sfGFP purification. B) Zeta-potential of sfGFP (-10) and sfGFP (+10) indicating the surface charge of each protein. DNA and amino acid sequences for sfGFP(-10) and (+10) are shown below. The altered amino acids for sfGFP are highlighted in red for positive changes and blue for negative changes.

sfGFP(-10)
ATGGGCCATCATCATCATCATCATCGCAGCAGCAAAGGCGAAGAACTGTTTACCGGCGTG
GTGCCGATTCTGGTGGAACTGGATGGCGATGTGAACGGCCATAAATTTAGCGTGCGCGGC
GAAGGCGAAGGCGATGCGACCAACGGCAAACTGACCCTGAAATTTATTTGCACCACCGGC
AAACTGCCGGTGCCGTGGCCGACCCTGGTGACCACCCTGACCTATGGCGTGCAGTGCTTT
AGCCGCTATCCGGATCATATGAAACGCCATGATTTTTTTAAAAGCGCGATGCCGGAAGGC
TATGTGCAGGAACGCACCATTAGCTTTAAAGATGATGGCACCTATAAAACCCGCGCGGAA
GTGAAATTTGAAGGCGATACCCTGGTGAACCGCATTGAACTGAAAGGCATTGATTTTAAA
GAAGATGGCAACATTCTGGGCCATAAACTGGAATATAACTTTAACAGCCATAACGTGTAT
ATTACCGCGGATGAACAGAAAAACGGCATTAAAGCGAACTTTAAAATTCGCCATAACGTG
GAAGATGGCAGCGTGCAGCTGGCGGATCATTATCAGCAGAACACCCCGATTGGCGATGGC
CCGGTGCTGCTGCCGGATAACCATTATCTGAGCACCCAGAGCGTGCTGAGCAAAGATCCG
GATGAAGAACGCGATCATATGGTGCTGCTGGAATTTGTGACCGCGGCGGGCATTACCCAT
GGCATGGATGAACTGTATAAA

MGHHHHHHRSSKGEELFTGVVPILVELDGDVNGHKFSVRGEGEGDATNGKLTLK FICTTGKLPVPWPT
LVTTLTYGVQCFSRYPDHMKRHDFFKSAMPEGYVQERTISFKDDGTYKTRAEVKFEGDTLVNRIELKGID
FKEDGNILGHKLEYNFNSHNVYITADEQKNGIKANFKIRHNVEDGSVQLADHYQQ NTPIGDGPVLLPDN
HYLSTQSVLSKDPDEERDHMVLLEFVTAAGITHGMDELYK

sfGFP(+10)
ATGGGCCATCATCATCATCATCATCGCAGCAGCAAAGGCGAAAAACTGTTTACCGGCGTG
GTGCCGATTCTGGTGGAACTGGATGGCGATGTGAACGGCCATAAATTTAGCGTGCGCGGC
GAAGGCGAAGGCGATGCGACCAACGGCAAACTGACCCTGAAATTTATTTGCACCACCGGC
AAACTGCCGGTGCCGTGGCCGACCCTGGTGACCACCCTGACCTATGGCGTGCAGTGCTTT
AGCCGCTATCCGAAACATATGAAACGCCATGATTTTTTTAAAAGCGCGATGCCGGAAGGC

TATGTGCAGGAACGCACCATTAGCTTTAAAAAAGATGGCACCTATAAAACCCGCGCGGAA
GTGAAATTTGAAGGCAAAACCCTGGTGAACCGCATTGAACTGAAAGGCATTGATTTTAAA
AAAAAAGGCAACATTCTGGGCCATAAACTGGAATATAACTTTAACAGCCATAACGTGTAT
ATTACCGCGGATAAAAAAAAAAACGGCATTAAAGCGAACTTTAAAATTCGCCATAACGTG
GAAGATGGCAGCGTGCAGCTGGCGGATCATTATCAGCAGAACACCCCGATTGGCAAAGGC
CCGGTGCTGCTGCCGGATAACCATTATCTGAGCACCCAGAGCGTGCTGAGCAAAGATCCG
AAAGAAAAACGCGATCATATGGTGCTGCTGGAATTTGTGACCGCGGCGGGCATTACCCAT
GGCATGGATGAACTGTATAAA

MGHHHHHHRSSKGEKLFTGVVPILVELDGDVNGHKFSVRGEGEGDATNGKLTLKFICTTGKLPVPWPT
LVTTLTYGVQCFSRYPKHMKRHDFFKSAMPEGYVQERTISFKKDGTYKTRAEVKFEGKTLVNRIELKGID
FKKKGNILGHKLEYNFNSHNVYITADKKKNGIKANFKIRHNVEDGSVQLADHYQQNTPIGKGPVLLPDN
HYLSTQSVLSKDPKEKRDHMVLLEFVTAAGITHGMDELYK

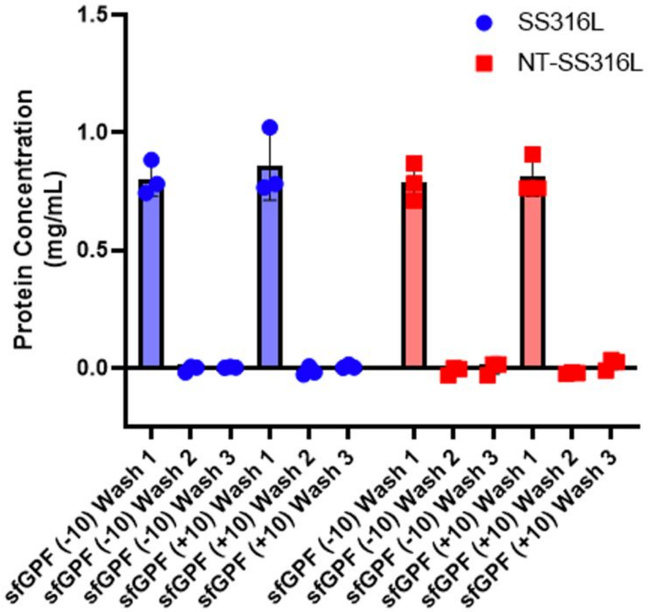

Figure S4. BCA assay measurement for soft corona removal from SS316L and NT-SS316L surfaces adsorbed with sfGFP (-10 and +10).

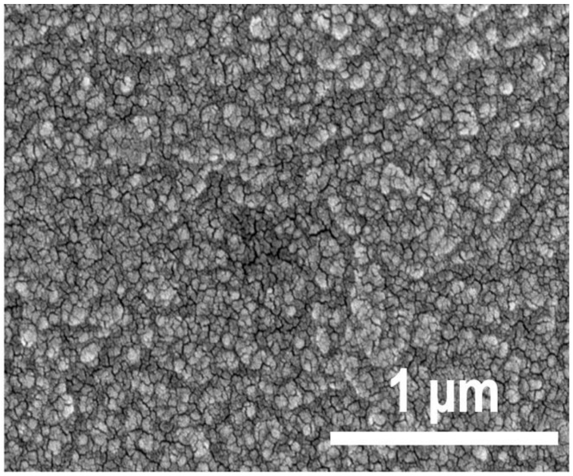

Figure S5. A) Scanning electron microscopy image of NT-SS316L surface architecture after protein adsorption, washing and dehydration. Scale bar is 1 µm.

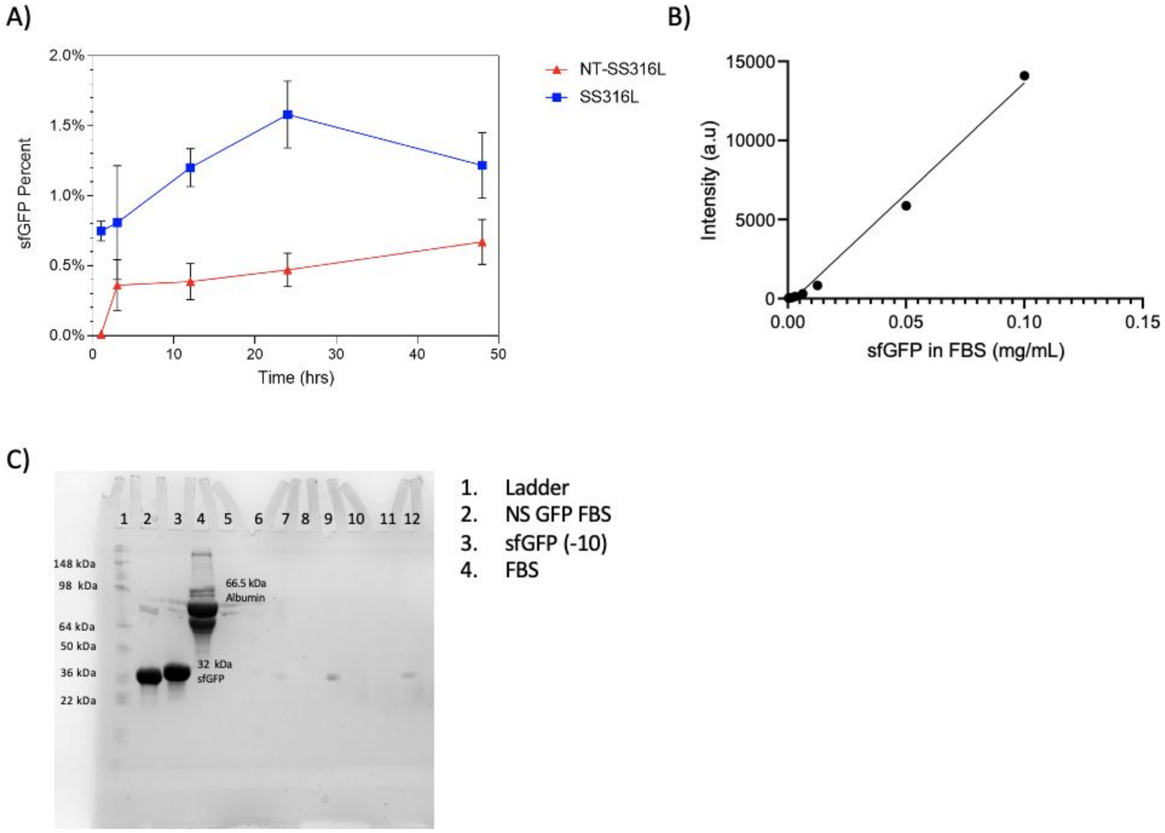

Figure S6. A) Percent of sfGFP(-10) protein adsorbed on the surfaces that is released in 10% FBS supplemented cell culture media. B) Calibration curve of sfGFP in 10% FBS media. C) SDS-PAGE of hard corona (lane 2) after 48 h. Comparison to pure sfGFP(-10) and FBS in lanes 3 and 4 indicate that the majority of the hard corona is sfGFP and very little serum protein adsorbed from the media.
